# Investigating the Genetic Etiology of Pediatric Patients with Peripheral Hypotonia Using the Next-Generation Sequencing Method

**DOI:** 10.1055/s-0042-1745873

**Published:** 2022-07-15

**Authors:** Damla Eker, Hakan Gurkan, Yasemin Karal, Sinem Yalcintepe, Selma Demir, Engin Atli, Serap T. Karasalihoglu

**Affiliations:** 1 Department of Medical Genetics, Faculty of Medicine, Trakya University, Edirne, Turkey; 2 Department of Pediatric Neurology, Faculty of Medicine, Trakya University, Edirne, Turkey

**Keywords:** hypotonia, peripheral hypotonia, next-generation sequencing, genetic etiology, multigene panel

## Abstract

**Background** Hypotonia occurs as a result of neurological dysfunction in the brain, brainstem, spinal cord, motor neurons, anterior horn cells, peripheral nerves, and muscles. Although the genotype–phenotype correlation can be established in 15 to 30% of patients, it is difficult to obtain a correlation in most cases.

**Aims** This study was aimed to investigate the genetic etiology in cases of peripheral hypotonia that could not be diagnosed using conventional methods.

**Methods** A total of 18 pediatric patients with peripheral hypotonia were included. They were referred to our genetic disorders diagnosis center from the Pediatric Neurology Department with a prediagnosis of hypotonia. A custom designed multigene panel, including *ACTA1* , *CCDC78* , *DYNC1H1* , *GARS* , *RYR1* , *COL6A1* , *COL6A2* , *COL6A3* , *FKRP* , *FKTN* , *IGHMBP2* , *LMNA* , *LAMA2* , *LARGE1* , *MTM1* , *NEM* , *POMGnT1* , *POMT1* , *POMT2* , and *SEPN1* , was used for genetic analysis using next-generation sequencing (NGS).

**Results** In our study, we found 13 variants including pathogenic (two variants in LAMA2) and likely pathogenic variants (three variants in RYR1 and POMGnT1) and variants of uncertain clinical significance (eight variants in RYR1, COL6A3, COL6A2, POMGnT1 and POMT1) in 11 (61%) out of 18 patients. In one of our patients, a homozygous, likely pathogenic c.1649G > A, p.(Ser550Asn) variant was defined in the *POMGnT1* gene which was associated with a muscle–eye–brain disease phenotype.

**Conclusion** The contribution of an in-house designed gene panel in the etiology of peripheral hypotonia with a clinical diagnosis was 5.5%. An important contribution with the clinical diagnosis can be made using the targeted multigene panels in larger samples.

## Introduction

Hypotonia occurs due to neurological dysfunction in the brain, brainstem, spinal cord, motor neurons, anterior horn cells, peripheral nerves, and muscles. 1 It is widely characterized by reduced muscle tone in the upper and lower extremities, the body, and craniofacial muscles. 2 While hypotonia is always observed in patients with muscle weakness, there may be no weakness in muscle strength in some cases of hypotonia. 2 3 4 It can be detected during birth or the neonatal period 5 and early childhood. 2 The incidence of peripheral hypotonia is unknown. 2

Any degree of dysfunction in the nervous system may cause hypotonia. 3 Hypotonia is etiologically divided into two groups depending on the location of the defect in the nervous system: peripheral and central hypotonia. 4 Peripheral hypotonia is caused by abnormalities in motor units, such as anterior horn cells (spinal muscular atrophy [SMA]) and peripheral nerves (neuropathies), neuromuscular connectivity diseases (botulism), and muscle diseases (myopathies). 3 With a frequency of 1/6,000 to 10,000, SMA is the most common cause of peripheral hypotonia, 6 followed by congenital myopathies and congenital myotonic dystrophies. 3 In peripheral hypotonia with lower motor neuron lesions, which constitute 15 to 30% of all hypotonic cases, genetic etiology presents different clinical features. Myopathies (5%), congenital myotonic dystrophies (4%), SMA (2%), muscular dystrophies (2%), and neuromuscular junction diseases (0.4%) are the genetic etiologies of hypotonia. 3 4 5

Next-generation sequencing (NGS) allows the design of targeted panels to identify etiology-related genes or candidate genes in patients and families in genetically heterogeneous conditions, such as neuromuscular diseases or unexplained hypotonia. NGS enables a faster genetic diagnosis with a reduced cost and sequencing time. 7 8 9 10 11

In our study, we aimed to identify the genetic etiology with NGS technology using an in-house designed multigene panel ( *ACTA1* , *CCDC78* , *DYNC1H1* , *GARS* , *RYR1* , *COL6A1* , *COL6A2* , *COL6A3* , *FKRP* , *FKTN* , *IGHMBP2* , *LMNA* , *LAMA2* , *LARGE1* , *MTM1* , *NEM* , *POMGnT1* , *POMT1* , *POMT2* , and *SEPN1* ) in cases with unexplained peripheral hypotonia using genetic methods such as conventional cytogenetics, array comparative genomic hybridization (array CGH), and Multiplex Ligation-dependent Probe Amplification (MLPA).

## Materials and Methods

### Patient Selection

Fifty-one patients (<8 years) with hypotonia and/or muscle weakness were referred to the Genetic Diseases Diagnosis Center from the Pediatric Neurology clinic between March 1, 2018, and November 1, 2019, were included. The written informed consent forms were obtained from legal guardians of the participants.

**Fig. 1:**
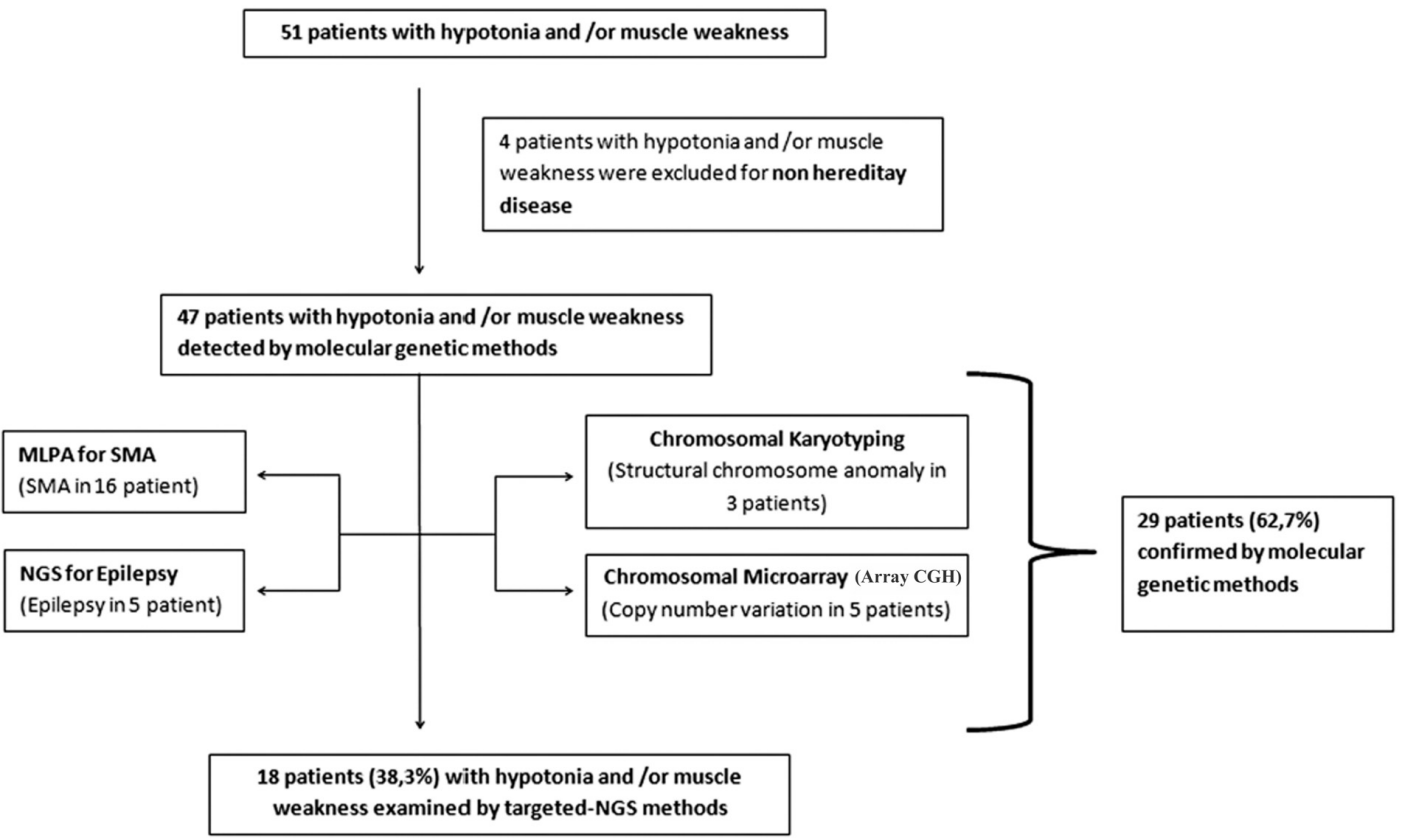
Workflow of genetic approach in patients with peripheral hypotonia. Array CGH, Array Comparative Genomic Hybridization; MLPA, Multiplex Ligation-dependent Probe Amplification; NGS, next-generation sequencing; SMA, spinal muscular atrophy.

A literature-supported diagnostic algorithm 7 10 was created to clinically evaluate patients with hypotonia and/or muscle weakness. Patients with birth trauma and brain anomaly, and patients diagnosed with other techniques in our algorithm were excluded from the study group ( Fig. 1 ). In total, 18 patients (38.3%) with unexplained peripheral hypotonia were included in the study by targeted-NGS method. The demographic characteristics and clinical findings of the patients are given in Table 1 . Ethics approval was obtained on February 2, 2018, from the Research Ethics Committee of our university with the number 02/05. This study was funded by the Scientific Research Projects Unit of our university with the project number 2018/268.

**Table 1:** Demographic characteristics and clinical findings of the patients

Patient	Sex	Age	Consanguinity	Clinical features	CK (U/L)	DTR
PHNGS-1	Female	2 years 7 months	The same village	Lower limb muscle weakness, delayed motor development	44	Absent
PHNGS-2	Male	2 years 4 months	No	Muscle weakness, lack of walking and crawling	58	Absent
PHNGS-3	Male	1 year 7 months	No	Muscle weakness, lack of walking, sitting and crawling, delayed motor development	95	Decreased
PHNGS-4	Male	2 years 2 months	1.5-degree cousin marriage	Muscle weakness, inability to walk, dysphagia, myoclonus, CK increase	8.185	Absent
PHNGS-5	Male	6 years 2 months	No	Lower limb muscle weakness, premature birth	94	Decreased
PHNGS-6	Female	8 months	No	Lower limb muscle weakness, delayed motor development, lack of head control and sitting	229	Decreased
PHNGS-7	Male	1 year 6 months	No	Lower limb muscle weakness, lack of walking and crawling	74	Decreased
PHNGS-8	Female	1 year 7 months	No	Polyhydramnios, delayed motor development	44	Absent
PHNGS-9	Male	1 year 5 months	First-degree cousin marriage	Lack of reflexes in the upper and lower extremities, delayed motor development	63	Absent
PHNGS-10	Female	1 year 4 months	No	Lack of walking, delayed motor development	76	Decreased
PHNGS-11	Female	4 years 9 months	Consanguinity?	Muscle weakness in the upper and lower extremities, delayed motor development	94	Decreased
PHNGS-12	Male	8 months	First-degree cousin marriage	Muscle weakness in the upper and lower extremities, lack of head control and sitting	50	Decreased
PHNGS-13	Male	1 year 8 months	First-degree cousin marriage	Muscle weakness in the upper and lower extremities, delayed motor development	612	Absent
PHNGS-14	Female	1 year 7 months	No	Muscle weakness in the upper and lower extremities, delayed motor development	56	Decreased
PHNGS-15	Male	5 years 1 months	Adopted child	Lower limb muscle weakness, delayed motor development	158	Decreased
PHNGS-16	Male	1 year 5 months	The same village	Lower limb muscle weakness, delayed motor development	138	Absent
PHNGS-17	Male	6 months	No	Lower limb muscle weakness	No	Decreased
PHNGS-18	Female	8 months	No	Delayed motor development, lack of walking and sitting	180	Decreased

Abbreviations: CK, creatin kinase; DTR, deep tendon reflex.

### Gene Selection and Panel Design

A total of 20 genes associated with peripheral hypotonia were selected. These genes were associated with or were candidate genes for motor neuron diseases, peripheral neuropathies, congenital muscular dystrophies (collagenopathies, dystroglycanopathies, and laminopathies), and congenital myopathies.

The selected genes consisted of 725 exons in total. All exons and splice sites of the genes were sequenced. The 15 genes, including *COL6A1* , *COL6A2* , *COL6A3* , *FKRP* , *FKTN* , *IGHMBP2* , *LMNA* , *LAMA2* , *LARGE* , *MTM1* , *NEB* , *POMGNT1* , *POMT1* , *POMT2* , and *SEPN1* , were prepared using the TruSight Rapid Capture sequencing panel with 552 genes designed to identify pediatric early-onset diseases (Illumina Inc., San Diego, California, United States). Five genes ( *ACTA1* , *CCDC78* , *DYNC1H1* , *GARS* , and *RYR1* ) were prepared using the QiaSeq specific amplicon panel (Qiagen, Hilden, Germany).

### DNA Isolation and Next-Generation Sequencing

Peripheral blood samples were introduced into an EDTA (ethylenediamine tetra-acetic acid) tube. Genomic DNA (gDNA) was prepared according to the manufacturer protocol of the EZ1 DNA isolation kit (Qiagen, Hilden, Germany) in the EZ1 advanced XL automated nucleic acid isolation system (Qiagen, Hilden, Germany). Concentrations and purity values of gDNAs were measured in NanoDrop (Thermo Scientific, United States). Specific codes (PHNGS) were given to each gDNA that passed the concentration and purity control and were stored at −20°C.

Libraries were prepared according to the manufacturer's instructions. The TruSight Rapid Capture sequencing panel (Illumina Inc., San Diego, California, United States) was used for 15 genes, and the QIAseq Targeted DNA panel (CDHS-15516Z-1355, Qiagen, Germany) was used for five genes with isolated gDNAs. Prepared libraries were sequenced using the NextSeq550 sequencing system (Illumina, San Diego, California, United States).

### Variant Filtering and Analysis

The FastQ data were aligned to the human genome sequence (Hg19/GRCh37). Illumina MiSeq software and Seq powered by Genomize were used for the analysis of the Trusight Rapid Capture kit, while the Qiagen Ingenuity Variant Analysis (IVA) and Qiagen Clinical Insight (QCI) were used to analyze the Qiaseq panel. The latest version of the Integrative Genomics Viewer (IGV) program was used to visually examine variants in relevant genes.

HGMD Professional 2020.1, ClinVar, dbSNP, MAF (minor allele frequency) and gnomAD 12 databases were used to determine whether the variation was associated with nucleotide changes. For in silico analysis, Mutation Taster, 13 polymorphism phenotyping v2 (PolyPhen2), 14 SIFT, 15 DANN (pathogenicity scoring methodology) scores, 16 combined annotation-dependent depletion (CADD) scores, 17 and genomic evolutionary rate profiling (Gerp) scores 18 were taken into consideration.

### Classifying the Variants

**Fig. 2:**
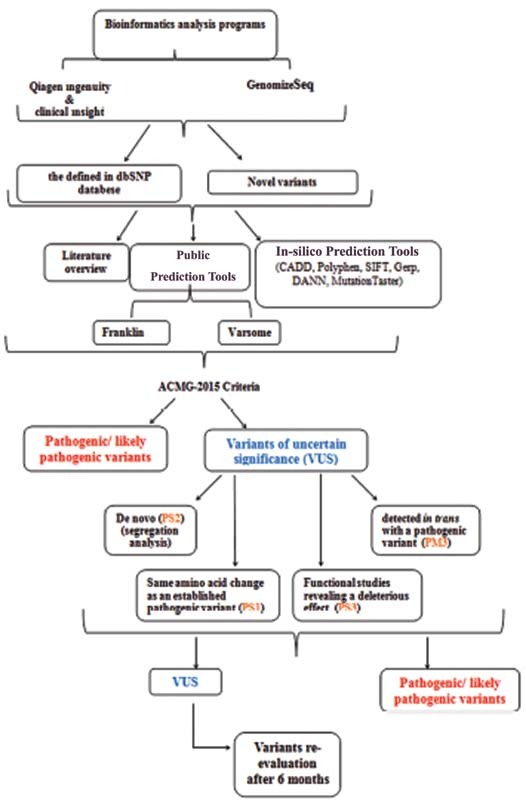
Workflow of variant filtering and classification. ACMG, American College of Medical Genetics; CADD, combined annotation-dependent depletion; Gerp, genomic evolutionary rate profiling; Polyphen, polymorphism phenotyping; VUS, variant of unknown significance.

Variants with an MAF value below 1% in the population were taken into consideration in filtering the variants. Allele frequencies of the variants were evaluated in the ExAC, GnomAD, GnomAD_Exom, and dbSNP public databases. Undefined variants were identified according to the Human Genome Variation Society (HGVS) guidelines, and variant pathogenicity was evaluated ( Fig. 2 ). The American College of Medical Genetics (ACMG)-2015 criteria were used to determine the pathogenicity of variants. 19

### Segregation Analysis with Sanger's Sequencing

A family segregation analysis was performed with in-house designed primer sets in 11 of the cases to evaluate the pathogenicity of the variants. The Big Dye Terminator Cycle Sequencing Kit v3.1 (Applied Biosystems, Foster City, California, United States) which contains fluorescent-labeled dideoxynucleotides that was used for bidirectional Sanger's sequencing. The amplicons prepared were sequenced with the Applied Biosystems ABI 3130XL Genetic Analyzer (Applied Biosystems, California, United States) device. The sequences obtained were evaluated using Proseq and BioEdit software programs.

## Results

The ages of the 18 participants with unexplained peripheral hypotonia ranged from 6 months to 7 years, and the average age was 2 years and 2 months. Seven of the 18 patients were female (38.89%) and 11 were male (61.11%). The average age of the females was 1 year and 11 months while that of the males was 2 years and 3 months.

Based on the neurological examination, 11 of the 18 patients (five females and six males) had a decrease in deep tendon reflex (DTR), while seven patients had absent DTRs (two females and five males).

The parents of five patients had consanguineous marriages, and the parents of two patients were from the same settlement. One patient was adopted, and the history relating to the consanguinity of the parents could not be evaluated.

In our study, we found 13 variants in 11 (61%) out of 18 patients. Two of these variants were evaluated as pathogenic, and they were both located in *LAMA2* . Three of them were likely pathogenic variants located in *RYR1* and *POMGnT1* genes, while eight variants were variants of unknown clinical significance located in *RYR1* , *COL6A2* , *COL6A3* , *POMGnT1* , and *POMT1* genes ( Table 2 ).

**Table 2:** Patients with pathogenic/likely pathogenic and variants of uncertain significance detected in our study

Patient	Gene	Mode	Transcript	HGVSc	Zygosity	HGVSp	Mutation type	Inheritance	Novel/reported	ACMG 2015	Pathogenicity According to ACMG 2015
PHNGS-1	*RYR1*	AD/AR	NM_000540.2	c.6670C > T	Heterozygous	p.(Arg2224Cys)	Missense	Paternal	rs199870223	PM2, PM5, PP3, PM1, PP2	Likely pathogenic
				c.3270G > A	Heterozygous	p.(Glu1090 = )	Synonymous	Maternal	rs577203385	PM2	Uncertain significance
PHNGS-3	*COL6A3*	AD/AR	NM_004369.3	c.6208C > T	Heterozygous	p.(Pro2070Ser)	Missense	Maternal	rs773478979	PM1, PM2, PP3	Uncertain significance
PHNGS-4	*POMGnT1*	AR	NM_017739.3	c.1649G > A	Homozygous	p.(Ser550Asn)	Missense	Parents heterozygous	rs193919335	PM2, PP2, PP5	Likely pathogenic
PHNGS-5	*COL6A3*	AD/AR	NM_004369.3	c.1688A > G	Heterozygous	p.(Asp563Gly)	Missense	Maternal	rs112913396	PP3	Uncertain significance
PHNGS-6	*LAMA2*	AR	NM_000426.3	c.3976C > T	Heterozygous	p.(Arg1326Ter)	Nonsense	Maternal	rs398123373	PVS1, PP5, PM2	Pathogenic
PHNGS-7	*LAMA2*	AR	NM_000426.3	c.7732C > T	Heterozygous	p.(Arg2578Ter)	Nonsense	Maternal	rs121913572	PVS1, PP5, PM2	Pathogenic
PHNGS-8	*COL6A2*	AD/AR	NM_001849.3	c.2182_2184delGTG insATA	Heterozygous	p.(Val728Ile)	Missense	–	–	–	–
PHNGS-11	*POMGnT1*	AR	NM_017739.3	c.766T > C	Heterozygous	p.(Trp256Arg)	Missense	Paternal	–	PM2, PP3	Uncertain significance
PHNGS-15	*RYR1*	AD/AR	NM_000540.2	c.14021G > A	Heterozygous	p.(Arg4674Gln)	Missense	Adopted child	rs1328709837	PM2, PP3, PP2	Uncertain significance
PHNGS-16	*COL6A3*	AD/AR	NM_004369.3	c.3266A > G	Heterozygous	p.(Gln1089Arg)	Missense	Maternal	–	PM2	Uncertain significance
	*POMT1*	AR	NM_007171.3	c.883G > A	Heterozygous	p.(Asp295Asn)	Missense	Paternal	rs754611085	PM2	Uncertain significance
PHNGS-18	*POMGnT1*	AR	NM_017739.3	c.1814G > A	Heterozygous	p.(Arg605His)	Missense	Paternal	rs267606962	PP3, PP5, PM2, PM5	Likely pathogenic

Abbreviations: ACMG, American College of Medical Genetics; AD, autosomal dominant, AR, autosomal recessive; HGVS, Human Genome Variation Society; HGVSc, Human Genome Variation Society Codon; HGVSp, Human Genome Variation Society Protein.

## Discussion

Peripheral hypotonia constitutes approximately 13 to 34% of all hypotonia cases (Paro-Panjan and Neubauer, [13%]; Laugel et al, [18%]; Birdi et al, [20%]; and Richer et al, [34%]). 20 21 22 23 There are different studies on the importance of algorithms in the etiological classification of hypotonia patients that cannot be defined by classical methods, including cases of central and peripheral hypotonia. 20 21 23 It is difficult to diagnose patients who cannot be identified with various algorithms and classical methods.

Peredo and Hannibal reported unexplained hypotonia in 13% of patients, 3 Paro-Panjan and Neubauer reported in 3% of patients, 20 and Birdi et al reported in 33% of their patients. 22 In our study, it was observed that 38.3% of patients with hypotonia/muscle weakness had unexplained hypotonia.

The NGS method is used for diagnosis because it is both effective and efficient in terms of time and cost in a nonspecific heterogeneous and complex disease group, such as hypotonia. 7 8 9 24

In our country, there are different studies on the identification of hypotonic cases using certain algorithms, 25 but there has been no study on the genetic etiology of unexplained peripheral hypotonia cases using the NGS method. Our work is the first study investigating the genetic etiology of hypotonia patients using NGS.

In our study, total 13 variants that classified as pathogenic, likely pathogenic, and variant of unknown significance (VUS) were detected in 11 out of 18 patients (61%). These variants were in *RYR1* (23.07%), *COL6A3 (* 23.07%), *COL6A2* (7.7%), *POMGnT1* (23.07%), *LAMA2* (15.38%), and *POMT1* (7.7%) genes.

Pathogenic or likely pathogenic variants in the *RYR1* gene have been associated with congenital myopathy, multi-mini core diseases and central core diseases, and have autosomal dominant and autosomal recessive inheritance. Davis et al and Amburgey et al defined three hotspot regions in *RYR1* gene mutations: (1) exons 2 to 7: N-terminal region, (2) exons 39 to 46: central region, and (3) exons 85 to 103: C-terminal region. 26 27 Wang et al identified pathogenic variants in nine different genes in 22 patients. 7 They reported that 32% of the pathogenic variants were detected in the *RYR1* gene. Chae et al reported that pathogenic/VUS variants in the *RYR1* gene are the most common. 8 Gonzalez-Quereda et al reported that the variants detected in the *RYR1* gene in 16 of 102 (15.7%) patients were clinically related. 11 In our study, three variants (3/13, 23%) were detected in the *RYR1* gene, and they were classified as likely pathogenic and VUS according to the ACMG-2015 classification in two patients. This result was consistent with the literature. The variant classified as likely pathogenic was located in the *RYR1* gene central region (exon 41, PHNGS1), and the variant classified as VUS was located in the C-terminal region of the *RYR1* gene (exon 96, PHNGS15).

In patient PHNGS1, two different variants were detected in the *RYR1* gene heterozygous c. 6670C > T and heterozygous c.3270G > A. One of the variants was inherited from the mother and one from the father in a trans position, verified by Sanger's sequencing. The in silico analysis results and the paternal inheritance of the c.6670C > T variant could not explain the clinical findings of patient PHNGS1.

In patient PHNGS15, the heterozygous c.14021G > A variant was detected in the *RYR1* gene. This variant is located in the C-terminal region of the protein encoded by the *RYR1* gene and is identified as rs1328709837 in the dbSNP database with a global allele frequency of 0.000008. 12 The variant detected in our study was not previously detected in studies related to neuromuscular diseases and hypotonia and was not reported in the ClinVar and HGMD professional 2020.1 databases. There are no functional studies related to the c.14021G > A variation. Segregation analysis of parents was not possible due to the adoption history of the patient. The c.14021G > A variant, which has not been previously reported in the literature and public databases, is compatible with clinical findings, and supporting its pathogenic potential instead of being classified as a VUS.

*POMGnT1* is the first reported gene associated with dystroglycanopathies. 28 Homozygous or compound heterozygous variants in *POMGnT1* have been associated with the muscle–eye–brain (MEB) disease phenotype. 29 30 MEB is an autosomal recessive disease characterized by congenital muscular dystrophy, eye abnormalities, and brain malformations. 31 Yoshida et al first described *POMGnT1* mutations in patients with MEB. 32 These mutations that cause loss of function or expression in *POMGnT1* cause hypoglycosylation of α-dystroglycan in the central nervous system and muscle, including in the brain and retina. 28 Taniguchi et al reported that while severe brain anomalies, such as hydrocephalus, were detected in patients with mutations close to the 5′ end of the *POMGnT1* gene, patients with a mutation close to the 3′ end had milder clinical findings in studies on the global distribution and clinical spectrum of MEB. 33 In our study, three different variants were found in the *POMGnT1* gene (3/13, 23%). One of these variants was homozygous and the remaining two were heterozygous.

In patient PHNGS4, a homozygous c.1649G > A variant was detected in the *POMGnT1* gene. This variant was reported as pathogenic in the study of Yoshida et al and has been associated with the MEB phenotype. 32 This variant was identified as rs193919335 in the dbSNP database. Its global allele frequency was reported as 0.000004, 12 and it was reported as likely pathogenic in the ClinVar database. In the segregation analysis performed in a consanguineous family, it was found that the mother and father carried the heterozygous c.1649G > A variant. In the OMIM database, *POMGnT1* pathogenic variants that exhibit autosomal recessive inheritance were associated with muscular dystrophy–dystroglycanopathy (congenital brain and eye anomalies), type A, and phenotype 3 which is one of the four phenotypes. In the anamnesis and physical examination of our patient who was 2 years and 2 months old, it was found that there was developmental retardation since birth, difficulty swallowing, the absence of DTRs, elevated creatin kinase (CK) enzyme, and noncontrast cranial magnetic resonance imaging (MRI) during the prenatal period. An ultrasonography performed during the postnatal period detected hydrocephalus. These findings supported the phenotype–genotype relationship in our patient. However, the presence of the c.1649G > A variant close to the 3′ end of *POMGnT1* in our patient, contradicted the results of Taniguchi et al. 33

Pathogenic and likely pathogenic variants in the *COL6A* , *COL6A1* , *COL6A2* , and *COL6A3* genes have been associated with the Ullrich congenital muscular dystrophy and Bethlem myopathy phenotypes, and they have autosomal dominant and autosomal recessive inheritance patterns. 34 35 The variability of clinical significance and complexity of molecular diagnosis in Ullrich's congenital muscular dystrophies is thought to be due to incomplete penetration and autosomal dominant or autosomal recessive mutations. 36 Briñas et al reported that de novo dominant mutations were seen in 61% of their patients. Fan et al reported that 62 pathogenic variants (34 of them de novo and 28 of them previously reported) were detected in their study in which they analyzed *COL6A* genes with Sanger's sequencing and NGS in 60 patients and their families to clinically diagnose muscular dystrophies characterized by muscle weakness and hypotonia. The genes with the most pathogenic variants were *COL6A2* , *COL6A1* , and *COL6A3* . 37 In our study, three variants (3/13, 23%) were found in the *COL6A3* gene in two patients and in the *COL6A2* gene in one patient. Variants detected in the *COL6A3* gene were evaluated as VUS according to ACMG-2015 criteria.

In patient, PHNGS8, the heterozygous c.2182_2184delGTGinsATA variant, was detected in the *COL6A2* gene in the bioinformatics analysis program. Based on the segregation analysis, it was concluded that the variant was not an indel but two separate variants. This result suggests that it is important to confirm the indel variations detected with Sanger's sequencing in the evaluation of NGS data (excluding reading errors due to repeat regions or high GC content and variants with low read depth). Based on the family segregation analysis performed using Sanger's sequencing, the c.2182G > A variant was inherited paternally, and the c.2184G > A variant was inherited both maternally and paternally. The allele frequency of the c.2184G > A variant was common polymorphism and was not clinically associated. The c.2182G > A variant did not explain the clinical findings of the patient due to the paternal inheritance despite the in silico results and low allele frequency. Incomplete penetrance 38 can be observed in the Ullrich congenital myopathy phenotype associated with the *COL6A* gene which exhibits an autosomal dominant inheritance pattern. 36 Although our patient's father had the same genotype, we suggest that the absence of clinical findings associated with myopathy might be related to the lack of penetrance. Functional studies are needed to clarify this information.

The targeted multiple gene panel had a 5.5% contribution rate to clinical diagnosis. Diagnosis rates varied in studies using multiple gene panels, and this difference may be due to the number of genes included in multiple gene panels. Diagnosis rates have been reported as 22.5% (35 genes) 7 and 48.8% (579 genes) 8 in two different studies using multiple gene panels. If the number of genes included is increased, the clinical diagnosis rate may increase.

## Conclusion

As a result, only a few patients were included in this study and, consequently, a low phenotype–genotype relationship was found. We suggest that a higher phenotype–genotype relationship can be determined with the NGS method if the gene numbers in targeted multigene panels are increased and different populations and large sample groups are used.
